# Design of an open-label extension trial of nerandomilast (BI 1015550) in patients with idiopathic pulmonary fibrosis and progressive pulmonary fibrosis (FIBRONEER™-ON)

**DOI:** 10.1186/s12890-025-03973-7

**Published:** 2025-12-04

**Authors:** Wim A. Wuyts, Luca Richeldi, Shervin Assassi, Arata Azuma, Vincent Cottin, Anna-Maria Hoffmann-Vold, Michael Kreuter, Justin M. Oldham, Fernando J. Martinez, Claudia Valenzuela, Marlies S. Wijsenbeek, Madhu Kanakapura, Alexandra James, Gerrit Weimann, Cyril Drzewuski, Carl Coeck, Toby M. Maher

**Affiliations:** 1 Department of Respiratory Medicine, Unit for Interstitial Lung Diseases, University Hospitals Leuven, Herestraat 49, Leuven, 3000 Belgium; 2 Unità Operativa Complessa di Pneumologia, Fondazione Policlinico Universitario A. Gemelli IRCCS, Università Cattolica del Sacro Cuore, Rome, Italy; 3 Division of Rheumatology, McGovern Medical School, University of Texas, Houston, TX USA; 4 Pulmonary Medicine and Oncology, Nippon Medical School, Tokyo, Japan; 5 Pulmonary Research Center, Mihara General Hospital, Saitama, Japan; 6 Hôpital Louis Pradel, Centre de Référence des Maladies Pulmonaires Rares, Hospices Civils de Lyon, UMR 754, INRAE, ERN-LUNG, Université Claude Bernard Lyon 1, Lyon, France; 7 Department of Rheumatology, Oslo University Hospital, Oslo, Norway; 8 Department of Pneumology, Mainz Center for Pulmonary Medicine, Mainz University Medical Center, Mainz, Germany; 9 Department of Pulmonary, Critical Care & Sleep Medicine, Marienhaus Clinic Mainz, Mainz, Germany; 10 Division of Pulmonary and Critical Care Medicine, University of Michigan, Ann Abor, MI USA; 11 Department of Medicine, Cornell University, New York City, NY USA; 12 ILD Unit, Pulmonology Department, Hospital Universitario de la Princesa, University Autonomade Madrid, Madrid, Spain; 13 Department of Respiratory Medicine, Erasmus Medical Center, Rotterdam, The Netherlands; 14 Boehringer Ingelheim Pharma GmbH & Co. KG, Ingelheim am Rhein, Germany; 15 Elderbrook Solutions GmbH, Bietigheim-Bissingen, Germany; 16 Boehringer Ingelheim International GmbH, Ingelheim am Rhein, Germany; 17 Boehringer Ingelheim, Reims, France; 18 Boehringer Ingelheim SComm, Brussels, Belgium; 19 Keck Medicine of USC, Los Angeles, CA USA; 20 National Heart and Lung Institute, Imperial College London, London, UK

**Keywords:** Idiopathic pulmonary fibrosis, Progressive pulmonary fibrosis, Open-label extension, Autoimmune disease interstitial lung disease, Nonspecific interstitial pneumonia

## Abstract

**Background:**

There is a need for more effective treatments for idiopathic pulmonary fibrosis (IPF) and progressive pulmonary fibrosis (PPF). Nerandomilast (BI 1015550), an oral preferential inhibitor of phosphodiesterase 4B, is being evaluated in two randomized Phase III trials: FIBRONEER™-IPF (NCT05321069) and FIBRONEER™-ILD (NCT05321082). FIBRONEER™-ON is an open-label extension (OLE) of these studies that will evaluate the long-term safety and efficacy of nerandomilast. Here, we describe the study design of the OLE.

**Methods:**

This prospective 98-week OLE will follow the Phase III parent trials, which are currently underway with 1177 patients enrolled in FIBRONEER™-IPF and 1178 patients enrolled in FIBRONEER™-ILD. Approximately 1700 patients from 44 countries are expected to complete the parent trials and will be eligible for continuing into the OLE; this estimate assumes that there will be a discontinuation rate of ~25% over the duration of the parent trials and > 90% of eligible patients will agree to participate in the OLE. Irrespective of whether previously on active treatment or placebo, all patients in the OLE will be treated with nerandomilast at either 9 mg or 18 mg twice daily, depending on which dose demonstrates the most favorable benefit–risk profile in the parent trials. The primary endpoint will be the occurrence of any adverse event over the course of the OLE. This trial will also monitor long-term efficacy outcomes, including forced vital capacity change, and time to first exacerbation, disease progression, hospitalization and death.

**Discussion:**

This trial will provide information on the long-term safety, tolerability and efficacy of nerandomilast in patients with IPF and PPF.

**Trial registration:**

FIBRONEER™-ON: ClinicalTrials.gov: NCT06238622, registered 2 February 2024. Protocol version and date: version 3.0, 29 Apr 2024. FIBRONEER™-IPF: ClinicalTrials.gov: NCT05321069, registered 11 March 2022.FIBRONEER™-ILD: ClinicalTrials.gov: NCT05321082, registered 11 March 2022.

**Supplementary Information:**

The online version contains supplementary material available at 10.1186/s12890-025-03973-7.

## Background

Idiopathic pulmonary fibrosis (IPF) is the archetypal progressive fibrosing interstitial lung disease (ILD) and is characterized by progressive fibrosis, worsening of lung function and dyspnea [1–4]. The median survival for untreated patients with IPF typically ranges from 2.5 to 3.5 years; however, the clinical course of individual patients can vary greatly from slow progression to acute exacerbation and death [5]. There are currently two approved antifibrotic therapies for the treatment of IPF: nintedanib [6, 7] and pirfenidone [8, 9].

Over 30% of patients with other mainly fibrosing ILDs may develop a progressive phenotype, known as progressive pulmonary fibrosis (PPF) [1, 3, 10–13]. PPF is characterized by worsening respiratory symptoms accompanied by physiologic and/or radiologic evidence of disease progression [1, 3]. Similar to patients with IPF, patients manifesting PPF have poor outcomes [1, 14]. The only antifibrotic therapy approved for the treatment of PPF is nintedanib [6, 7, 15]. In the RELIEF trial, treatment with pirfenidone was associated with significantly lower decline in forced vital capacity (FVC) percent predicted compared with placebo in patients with PPF, but due to methodology and recruitment issues, the evidence has been deemed as low and it has since not been approved for the treatment of PPF [16]. Additionally, nintedanib and tocilizumab are approved in the United States (along with rituximab in Japan and nintedanib in the EU) for slowing the rate of decline in pulmonary function in patients with systemic sclerosis-associated ILD (SSc-ILD) [6, 17, 18].

The antifibrotic treatments approved for IPF and PPF can slow down, but do not stop or reverse, disease progression, and are associated with side effects that can delay treatment or lead to discontinuation, highlighting an unmet need for additional treatments [10, 19, 20].

Phosphodiesterase 4 (PDE4) is widely expressed in immune system cells, and its inhibition is associated with broad anti-inflammatory and antifibrotic properties [19]. Historically, the use of oral pan-PDE4 inhibitors in different diseases has been limited due to adverse events (AE) [10, 19, 21]. However, it has been suggested that preferentially inhibiting PDE4B may be associated with improved tolerability compared with pan-PDE4 inhibitors [10, 11].

Nerandomilast (BI 1015550) is a preferential inhibitor of PDE4B. In Phase I and II studies, nerandomilast appeared to have an acceptable safety and tolerability profile [10, 11, 19, 22]. Furthermore, in a Phase II trial in patients with IPF, nerandomilast prevented lung function decline over 12 weeks, either alone or in combination with background antifibrotic therapy [22]. As a result, the efficacy and safety of nerandomilast is now being investigated in the Phase III FIBRONEER™ clinical trials, FIBRONEER™-IPF and FIBRONEER™-ILD, in patients with IPF and PPF respectively, in the form of 9 mg and 18 mg twice-daily dose (with and without background antifibrotics) over 52 weeks [10, 20].

This manuscript describes the design of the FIBRONEER™-ON trial, the open-label extension (OLE) of the two ongoing parent trials, FIBRONEER™-IPF (NCT05321069) and FIBRONEER™-ILD (NCT05321082). This OLE will evaluate the long-term safety, tolerability, and efficacy of nerandomilast at either 9 mg or 18 mg twice daily in patients with IPF and PPF.

## Methods

### Trial design

**Fig. 1:**
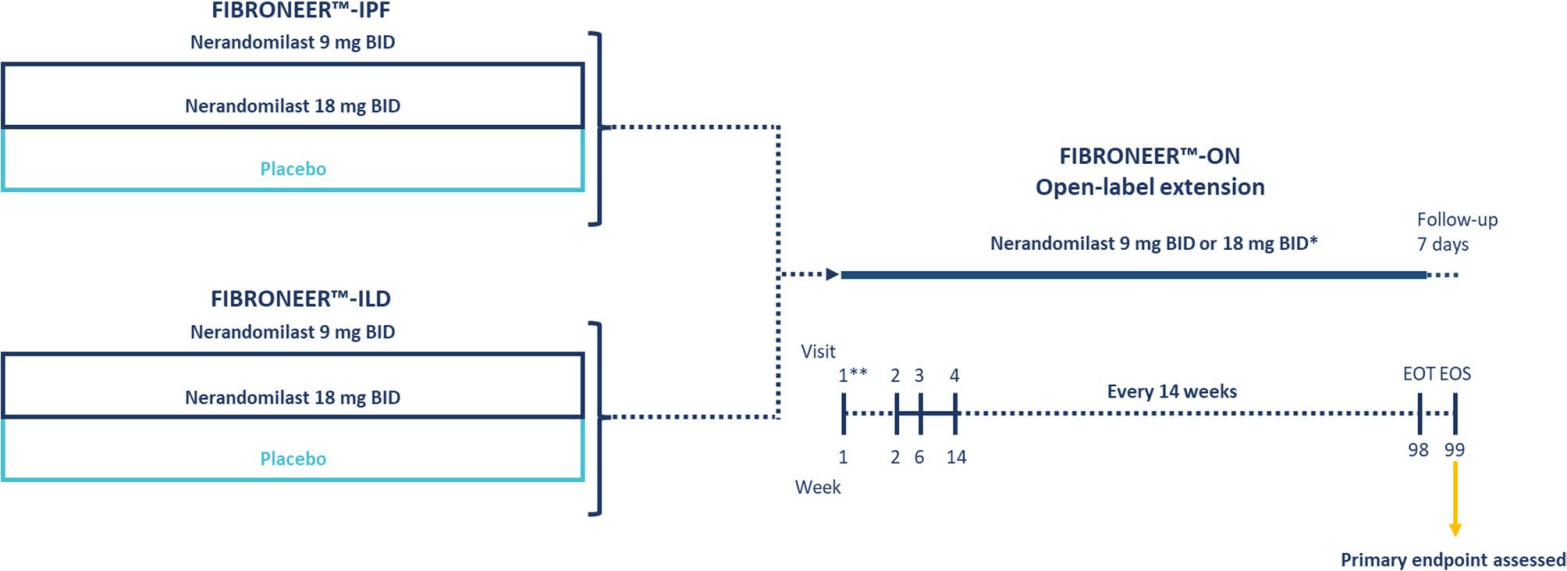
Trial design. *Patients will receive nerandomilast at either 9 mg or 18 mg BID, depending on which dose provides the most favorable benefit–risk profile in the parent trial. **The first visit may occur on the last day of the parent trial. *BID* twice daily; *EOS* end of study; *EOT* end of treatment; *ILD* interstitial lung disease; *IPF *idiopathic pulmonary fibrosis

This Phase III, multicenter, multinational, prospective OLE will be conducted if there is a positive benefit–risk outcome from the evaluation of the Phase III parent trials (Fig. 1). The parent trials are currently underway in 44 countries, with 1177 patients enrolled in FIBRONEER™-IPF and 1178 patients enrolled in FIBRONEER™-ILD. FIBRONEER™-IPF and FIBRONEER™-ILD are estimated to complete at the end of 2024 and beginning of 2025, respectively [10, 20, 23, 24]. Approximately 1700 patients with IPF or PPF will be eligible for FIBRONEER™-ON, assuming a discontinuation rate of ~25% over the duration of the parent trials and >90% of eligible patients agreeing to participate in this trial.

The primary analysis of the parent trials will be performed when the last patient reaches Week 52. Once the data becomes available and a positive benefit–risk assessment is reached, the OLE will be opened using the most favorable dose. All eligible patients will have the option to roll-over into FIBRONEER™-ON. Treatment start in the OLE will occur without interruption wherever possible. In the case where roll-over without interruption is not possible, eligible patients can start participation and treatment in the OLE with a maximum interruption of 12 weeks between their respective parent trial and the OLE.

Patients will receive treatment at a dose of 9 mg or 18 mg twice daily (whichever dose showed the most favorable benefit–risk profile in parent trials), with treatment duration not exceeding 98 weeks. Approximately 1 in 3 patients that will enter the OLE will switch from placebo to active treatment. A follow-up period of a minimum of 7 days (+ 3-day window) after end of treatment (EOT) will be required. All patients will complete a visit at the end of the follow-up period for AE assessment, recording of concomitant medication, and assessment of mental health status.

A clinical trial leader will be responsible for managing the trial in accordance with applicable regulations and internal standard operating procedures; directing the clinical trial team in the preparation, conduct, and reporting of the trial; and ensuring appropriate training and information for clinical trial managers, clinical research associates, and investigators of participating countries. All assessments will be carried out by clinical trial investigators.

### Inclusion and exclusion criteria

Patients with IPF or PPF who complete the parent trials on blinded treatment (i.e., who do not prematurely discontinue trial treatment permanently in parent trials) and provide written informed consent, are allowed to participate in this OLE.

Stem cell therapy and PDE4 inhibitors other than nerandomilast remain restricted in FIBRONEER™-ON. Other main inclusion and exclusion criteria can be found in Table 1, with further inclusion and exclusion criteria in Supplementary Table 1.

**Table 1:** Main inclusion and exclusion criteria

Main inclusion criteria	
• Patients who completed treatment in the parent trials (FIBRONEER™-IPF or FIBRONEER™-ILD) without prematurely discontinuing treatment permanently according to protocol (i.e., completed treatment with or without temporary treatment interruption) • Signed and dated written informed consent in accordance with ICH-GCP and local legislation prior to admission to the trial	

Main exclusion criteria	
• Any disease that may put the patient at risk when participating in this trial, at the investigator’s discretion • Patient exhibits suicidality in the clinical judgment of the investigator or according to the following criteria at Visit 1: • any suicidal behavior (i.e., actual attempt, interrupted attempt, aborted attempt, or preparatory acts or behavior) • any suicidal ideation of type 4 or 5 in the C-SSRS (i.e., active suicidal thought with intent but without specific plan, or active suicidal thought with plan and intent) • Patients with clinically relevant severe depression at investigator’s discretion or a HADS sub-score >14 at Visit 1 • An occurrence of malignant neoplasm other than appropriately treated basal cell carcinoma or in situ squamous cell carcinoma of the skin or in situ carcinoma of uterine cervix at Visit 1 • Patients with a BMI <18.5 kg/m² that experienced an additional, unexplained and clinically significant (>10%) weight loss during the parent trials • Patient will undergo lung transplantation • At Visit 1, patients with ongoing AESI (suspected vasculitis, DILI, severe infections) that led to temporary treatment interruption in the parent trials • Patients who must or wish to take restricted medications such as stem cell therapy or PDE4 inhibitors^a^ and non-selective PDE inhibitors^b^ or any drug considered likely to interfere with the safe conduct of the trial	

^a^Roflumilast, apremilast, crisaborole, drotaverine

^b^Theophylline, aminophylline, oxtriphylline, dyphylline, pentoxifylline, ibudilast, tofisopam, dipyridamole

*AESI* Adverse event of special interest, *BMI* Body mass index, *C-SSRS* Columbia-Suicide Severity Rating Scale, *DILI* Drug-induced liver injury, *GCP* Good clinical practice, *HADS* Hospital Anxiety and Depression Scale, *ICH* International Council on Harmonization of Technical Requirements for Pharmaceuticals for Human Use, *PDE* Phosphodiesterase.

### Ethics approval

This OLE will be carried out in compliance with the protocol, the ethical principles laid down in the Declaration of Helsinki, in accordance with the International Council for Harmonization Guidelines for Good Clinical Practice (GCP), the European Union (EU) directive 2001/20/European Commission/EU regulation 536/2014, the Japanese GCP regulations, and other relevant regulations.

### Endpoints

The primary endpoint is whether a patient had any AE over the course of the extension trial (yes/no) i.e. up until the follow-up (FU)/end of study (EOS) visit planned at Week 99. Secondary and further endpoints include the absolute change from baseline in FVC (mL) and in percent predicted FVC over time; time to first acute IPF/PPF exacerbation, first hospitalization for respiratory cause, or death; and absolute change from baseline in Living with Pulmonary Fibrosis (L-PF) Symptoms in dyspnea, cough, and fatigue domain scores over time. All other secondary endpoints can be found in Table 2, with further endpoints in Supplementary Table 2.

**Table 2:** Primary and secondary endpoints

Primary endpoint	
• Whether patient had any adverse event over the course of the extension trial (yes/no), i.e. up until the follow-up/end of study visit planned at Week 99	

Secondary endpoints	
• Absolute change from baseline in FVC (mL) and in % predicted FVC over time • Time to absolute decline in FVC % predicted of >10% from baseline over the duration of the trial • Time to first acute IPF/PPF exacerbation, first hospitalization for respiratory cause, or death (whichever occurs first) over the duration of the trial • Time to first acute IPF/PPF exacerbation or death over the duration of the trial • Time to hospitalization for respiratory cause or death over the duration of the trial • Time to absolute decline in FVC % predicted of >10% from baseline or death over the duration of the trial • Time to relative decline in FVC % predicted of >10% from baseline or death over the duration of the trial	

*FVC* Forced vital capacity, *IPF* Idiopathic pulmonary fibrosis, PPF Progressive pulmonary fibrosis.

### Assessments of safety

Data and information necessary for the assessment of AEs, serious AEs, and AEs of special interest (AESIs) will be reported via electronic case report forms (CRFs) by trial investigators. AESIs include potential severe drug-induced liver injury, vasculitis, serious opportunistic or *Mycobacterium tuberculosis* infections, new onset of severe depression (defined as Hospital Anxiety and Depression Scale [HADS] sub-score >14), and new onset of severe anxiety (defined as HADS sub-score >14). Other safety parameters include psychological monitoring for suicidal risk and depression and anxiety, using the Columbia-Suicide Severity Rating Scale (C-SSRS) and HADS, as previously mentioned.

Physical examinations, vital signs, resting 12-lead electrocardiogram, routine safety laboratory tests (hematology, clinical chemistry, infectious serology, urinalysis, and immunologic vasculitis markers) will be taken at the beginning of the trial and at predetermined time points throughout the trial. All analyses will be performed by a central laboratory that will send reports to the investigator and study sponsor.

### Management of AEs

Patients experiencing diarrhea can be provided with counseling, including dietary advice, monitoring of adequate hydration, and potential treatment with anti-diarrheals, such as loperamide, that are used in routine clinical practice.

For patients on antifibrotic medication, management strategies (e.g., dose reduction and/or temporary interruption) as described in the respective prescribing information should be followed.

In case of events suspicious for a new onset of vasculitis, trial treatment will be discontinued, and the patient will be monitored. However, if the required thorough diagnostic workup shows no relationship between the trial treatment and the event, patients may resume their trial medication.

For patients experiencing severe depression, referral to a psychiatrist is recommended and trial treatment will be discontinued. However, if no causal relationship between the medication and symptoms are found, and the patient has recovered, they may resume the trial treatment based on the investigator’s judgment. For patients experiencing any suicidal behavior of type 4 or 5 on the C-SSRS during the trial, trial treatment will be permanently discontinued.

All efforts should be made to re-start treatment after resolution of AEs according to the investigator’s assessment.

### Assessments of efficacy

Based on the similarity between IPF and PPF, this OLE will adapt the most recent definition of acute exacerbations in IPF/PPF as follows [25]: acute exacerbation of IPF/PPF will be defined as an acute, clinically significant respiratory deterioration characterized by evidence of new widespread alveolar abnormality with all of the following, as assessed by the investigator: acute worsening or development of dyspnea typically less than 1 month in duration; high-resolution computed tomography (HRCT) with new bilateral ground-glass opacity and/or consolidation superimposed on a background pattern consistent with fibrosing ILD; and respiratory deterioration not fully explained by cardiac failure or fluid overload. Hospitalization due to respiratory cause will be collected on a specific non-elective hospitalization CRF page. The CRF page will capture the date of hospitalization, whether the non-elective hospitalization was due to respiratory cause, and the primary admission diagnosis.

FVC will be measured using spirometry measurements according to American Thoracic Society/European Respiratory Society guidelines.

To assess health-related quality of life in patients over the duration of the OLE, patients will be required to complete a L-PF-44 questionnaire. This questionnaire consists of 44-items covering symptoms (dyspnea, cough, and fatigue; 23 items) as well as impact on quality of life (21 items).

### Discontinuation of treatment

When a patient discontinues trial medication permanently, EOT visit activities will be performed at the time of discontinuation and a FU/EOS visit will be performed 7 days (+ 3-day window) after the last intake of trial medication.

All abnormal values (including laboratory parameters and AEs) at the time of an individual patient’s EOT that are judged clinically relevant by the investigator will be monitored using the appropriate tests until resolved. All AEs persisting after an individual patient’s EOS visit must be followed up until they have either been resolved or assessed as ‘chronic’ or ‘stable’, or no further information can be obtained. Temporary treatment interruption of nerandomilast will be permitted to manage AEs, infections, temporary treatment with restricted medications, or for other medical reasons, such as surgery. Reintroduction of treatment will be encouraged based on the judgment of the investigator.

Patients must discontinue treatment if they are repeatedly shown to be non-compliant with important trial procedures and, in the opinion of both the investigator and sponsor representative, the safety of the patient cannot be guaranteed as he/she is not willing or able to adhere to the trial requirements in the future.

### Exploratory endpoints

Exploratory blood biomarkers will be investigated and correlated to clinical endpoints. This includes protein biomarkers indicative of epithelial damage and fibrotic remodeling such as Krebs von den Lungen-6, matrix metalloproteinase-7, and surfactant protein D, and neoepitope markers such as neoepitope of type III collagen, neoepitope of type VI collagen, and propeptide of type III collagen [26–28]. An optional HRCT scan can be performed at the start of the OLE (within a 2-week time window) for quantitative assessment of fibrotic features, which will be compared with the baseline HRCT of the parent trial.

### Statistical methods

The primary endpoint will be analyzed using the number and percentage of patients with an AE over the duration of the OLE. The percentage will be provided with Wilson-type 95% confidence intervals. The absolute change from baseline in FVC (mL) and FVC percent predicted will be analyzed using descriptive statistics by visit. Mean changes from baseline in FVC will be accompanied by 95% confidence intervals. Time-to-event endpoints will be analyzed and presented with the proportion of patients with an event of interest over the trial, both descriptively and by Kaplan–Meier estimates with confidence intervals (using Greenwood variance formula). Kaplan–Meier plots will be presented as both pooled data and by randomized treatment groups in the parent trials. The absolute change from baseline in patient-reported outcome scores will be analyzed using descriptive statistics by visit over the duration of the OLE trial. Primary outcome assessments will also be analyzed by subgroups of the parent trials, including baseline antifibrotic or immunomodulatory treatment. As per protocol, an interim analysis is planned after 1 year or could be performed upon request from health authorities.

### Data handling

The trial sites will retain the source and essential documents according to contract or the local requirements valid at the time of the end of the trial (whichever is longer), at least for 25 years for EU countries. Data protection and data security measures are implemented for the collection, storage, and processing of trial participant data in accordance with principles 7 and 12 of the World Health Organization GCP handbook.

A quality assurance audit/inspection of this trial may be conducted by the sponsor, sponsor’s designees, or by an Institutional Review Board/Independent Ethics Committee or regulatory authority. The quality assurance auditor will have access to all medical records, the investigator’s trial-related files and correspondence, and the informed consent documentation of this clinical trial.

Missing or incomplete AE dates will be imputed according to sponsor rules, using an algorithm based on the worst-case approach. The imputed AE onset date will maximize the possibility for the AE to be counted as treatment emergent. No imputation is planned for other safety criteria. Missing or incomplete data for survival are managed by censored data analyses; no specific procedures need to be specified to handle them. For spirometry endpoints, missing data will not be imputed.

## Discussion

The FIBRONEER™-ON OLE will allow the safety, tolerability, and efficacy of the preferential PDE4B inhibitor nerandomilast to be investigated in patients with IPF and PPF over a longer duration compared with the parent trials. In addition, the OLE trial will allow patients to continue on a treatment with a favorable benefit–risk profile after the conclusion of the parent trials.

The antifibrotic treatments approved for IPF and PPF can slow down, but do not stop or reverse, disease progression, and are associated with side effects that can delay treatment or lead to discontinuation, highlighting an unmet need for additional treatment [10, 19, 20]. Preferential PDE4B inhibition by nerandomilast has a dual mode of action with antifibrotic and immunomodulatory effects. It is anticipated to have similar treatment effects in patients with IPF and PPF [10, 11, 20].

The FIBRONEER™ trials are investigating the efficacy and safety of nerandomilast in patients with IPF and PPF over 52 weeks. In the Phase II trial, 18 mg nerandomilast twice daily, either alone or in combination with background antifibrotic therapy, prevented lung function decline in patients with IPF. This supported the selection of the 18 mg twice-daily dose in the FIBRONEER™ trials; however, an additional 9-mg dose is being used to provide further dose-response and exposure-response data [10, 20, 22]. Depending on the results of the FIBRONEER™ trials, patients in FIBRONEER™-ON will be treated with either nerandomilast 9 mg or 18 mg twice daily.

There have been no previous clinical trials of nerandomilast in patients with PPF. However, many similarities have been observed between IPF and PPF in terms of disease progression, genetics, risk factors, response to therapy, and mortality [10, 29–33]. Moreover, the results of nerandomilast in a silica-induced lung fibrosis model in mice (tentatively representing the human counterpart of a progressive fibrosing ILD) achieved dose-dependent improvements in semi-quantitative scores of granuloma formation, inflammation, and fibrosis [11]. Collectively, the preclinical and clinical data support the rationale for investigating nerandomilast as a potential treatment for patients with PPF [10, 11, 19, 22].

### Rationale for endpoints

The primary endpoint for the parent trials is the absolute change from baseline in FVC (mL) at Week 52 [10, 20]. As the focus of this OLE will be on long-term safety and tolerability, the primary endpoint is whether a patient had any AE over the course of the OLE. In the Phase II trial in patients with IPF, diarrhea was the most frequent AE in nerandomilast. While the parent trials will determine AEs related to nerandomilast versus placebo, this OLE will provide data on the long-term tolerability of gastrointestinal side effects, with and without background antifibrotic treatment [10, 20, 22].

Change in FVC is a commonly used endpoint in ILD trials that have been associated with mortality [3]. To investigate the long-term efficacy of nerandomilast in this OLE, absolute change in FVC has been included as a secondary endpoint.

A challenge in IPF and PPF clinical trials is determining appropriate patient-reported outcome measures (PROMs) to capture change in quality of life over time [34]. Similar to the parent trials, L-PF-44 is being used in FIBRONEER™-ON; L-PF is a validated PROM that can be used in patients with IPF [35] and was found to best capture the signs/symptoms and impacts in a consensus study on PROMs for IPF and PPF [36].

### Monitoring of adverse events of special interest

Pan-PDE4 inhibitors have previously been associated with a number of AEs, such as infections (including severe and opportunistic), vasculitis (preclinical), depression, and suicidal ideation [20, 37–45]. As such, these will be AESIs in this trial. In Phase I trials with nerandomilast, no AEs of vasculitis, depression, suicidal ideation or behavior were reported [19]. In the Phase II trial of nerandomilast, serious infections were balanced between control and treatment groups [22]; nasopharyngitis was more frequently reported in the treatment group of the Phase Ic trials, but the numbers were very small [19].

### Differences between this OLE and the parent trials

In this OLE, a HRCT scan can be performed at the start of the OLE (within a 2-week time window) for quantitative assessment of fibrotic features, which will be compared with the baseline HRCT of the parent trial. In the parent trials, patients are required to have a HRCT scan to determine study eligibility [10, 20]. However, the scans in this OLE will be optional at certain sites and countries.

In this OLE, there are no restrictions for treatment of the underlying disease or other treatments for IPF/PPF (other than other PDE4 inhibitors).

Patients in FIBRONEER™-IPF are permitted to take background antifibrotics provided they were on stable treatment for 12 weeks prior to the first visit and were not on a combination of nintedanib and pirfenidone. Immunomodulatory medications other than prednisone ≤15 mg/day or equivalent for respiratory or pulmonary reasons are not permitted [20].

In FIBRONEER™-ILD, patients are permitted to take background nintedanib treatment provided they were on stable treatment for 12 weeks prior to randomization. They are also permitted to take immunosuppressive agents for an underlying systemic disease, including methotrexate and azathioprine, but not oral corticosteroids (>15 mg/day) within 4 weeks, cyclophosphamide, tocilizumab, or mycophenolate within 8 weeks, or rituximab within 6 months before Visit 1. As per the protocols of both parent trials, the use of cytochrome 3A4 or other PDE4 inhibitors is not permitted [10, 20].

The greater restriction of medication in the parent trials, such as steroids and immunosuppressive agents (specifically mycophenolate), could be seen as a limitation. Although limiting immunosuppressive agents in these studies is to avoid confounding variables, it is less reflective of a real-world population, as many patients with PPF are on immunosuppressants [10, 46]. With fewer restrictions of medication in FIBRONEER™-ON, it will allow data to be collected on the safety, tolerability, and efficacy of nerandomilast that is more similar to the real world, where patients are typically on multiple background medications. This could help inform clinical guidance for patients with PPF, particularly in patients with connective tissue disease-associated ILD.

Finally, unlike previous OLEs in IPF and PPF, FIBRONEER™-ON will combine patients from both parent trials, meaning patients with both IPF and PPF will be included. This will give additional insights into the effect of preferential PDE4B inhibition on different patient populations within the same study. As a result, it will reduce the associated limitation of heterogeneity as seen in other OLE studies that have a less diverse patient population.

### Limitations

Limitations of the OLE include the open-label nature of the study—including a population that has already shown toleration of the experimental drug from the parent trials—meaning the safety and tolerability of the drug in a real-world population may differ. Additionally, the absence of a placebo control limits the robustness of the safety analysis. OLEs are also not appropriate for assessing efficacy. Finally, pretreatment and longer disease duration in these patients may impact further disease progression and tolerability of treatments.

## Conclusions

The two FIBRONEER™ trials are the first Phase III trials investigating a preferential PDE4B inhibitor in IPF and PPF. FIBRONEER™-ON is anticipated to have the first patient enrolled in September 2024. The OLE will increase our understanding of the long-term safety, tolerability, and efficacy of nerandomilast in patients with IPF and PPF, and will provide a better understanding of the agent in daily clinical practice over an extended period of time.

## Supplementary Information

Supplementary Material 1.

Supplementary Material 2.

## Data Availability

To ensure independent interpretation of clinical trial results and enable authors to fulfil their role and obligations under the ICMJE criteria, Boehringer Ingelheim grants all external authors access to relevant clinical trial data. In adherence with the Boehringer Ingelheim Policy on Transparency and Publication of Clinical Trial Data, scientific and medical researchers can request access to clinical trial data, typically, one year after the approval has been granted by major Regulatory Authorities or after termination of the development program. Researchers should use the https://vivli.org/ to request access to trial data and visit https://www.mytrialwindow.com/msw/datasharing for further information.

## Abbreviations

AE: Adverse event
AESI: Adverse events of special interest
CRF: Case report form
C-SSRS: Columbia-Suicide Severity Rating Scale
EOS: End of study
EOT: End of treatment
EU: European Union
FU: Follow-up
FVC: Forced vital capacity
GCP: Good clinical practice
HADS: Hospital Anxiety And Depression Scale
HRCT: High-resolution computed tomography
ILD: Interstitial lung disease
IPF: Idiopathic pulmonary fibrosis
L-PF: Living with Pulmonary fibrosis
OLE: Open-label extension
PDE4: Phosphodiesterase 4
PPF: Progressive pulmonary fibrosis
PROM: Patient-reported outcome measures
SSc: Systemic sclerosis
