# Supplementary material for: Design of an open-label extension trial of nerandomilast (BI 1015550) in patients with idiopathic pulmonary fibrosis and progressive pulmonary fibrosis (FIBRONEER™-ON)
Source: BMC Pulm Med. 2025 Dec 4;26:10. doi: 10.1186/s12890-025-03973-7 (PMC12797674; doi:10.1186/s12890-025-03973-7)
Supplement: Supplementary file 1 — Supplementary Material 1. [file 12890_2025_3973_MOESM1_ESM.pdf]

Why is nerandomilast being investigated in idiopathic pulmonary fibrosis (IPF) and progressive pulmonary fibrosis (PPF)?

- **IPF** and **PPF** are rare lung diseases that cause inflammation and lung scarring that gets worse over time
- **Nerandomilast** is a new molecule under investigation in clinical trials that **blocks the activity of PDE4B** (a type of protein) and **reduces inflammation and scarring** of the lungs
- There are two ongoing parent trials – **FIBRONEER™-IPF** and **FIBRONEER™-ILD** – that are investigating nerandomilast for how safe and effective it is at treating patients with IPF and PPF over at least **52 weeks**
- **FIBRONEER™-ON** is an **extension trial** of FIBRONEER™-IPF and FIBRONEER™-ILD

Nerandomilast 9 mg

OR

Nerandomilast 18 mg

OR

Placebo

Who is taking part in FIBRONEER™-ON?

- Patients who have completed **FIBRONEER™-IPF** and **FIBRONEER™-ILD**
- **FIBRONEER™-ON** is anticipated to begin **September 2024.** Roughly 1,700 patients with **IPF** and **PPF** will take part
- Unlike in the parent trials, patients may take more medications that are commonly prescribed for IPF and PPF

Study medication

Study participants

FIBRONEER™-ON: An open-label extension trial of nerandomilast in patients with IPF and PPF

Study outcomes

Study design

What is FIBRONEER™-ON looking at?

The trial will assess the **long-term safety and efficacy** of nerandomilast in patients with IPF and PPF

Assessments of safety

- Physical examination
- Vital signs
- Safety laboratory tests
- Electrocardiogram
- Mental health monitoring*
- Monitoring of adverse events/ side effects

What does FIBRONEER™-ON involve?

The extension trial will begin at the **end of the parent trials**

Patients will take either **9 mg** or **18 mg** nerandomilast tablet **twice daily** (depending on the best dose in the parent trials)

FIBRONEER™-IPF
52 weeks

Nerandomilast 9 mg

Nerandomilast 18 mg

Placebo

FIBRONEER™-ILD
52 weeks

Nerandomilast 9 mg

Nerandomilast 18 mg

Placebo

FIBRONEER™-ON
98 weeks

Nerandomilast 9 mg

OR

Nerandomilast 18 mg

During the first **14 weeks** of treatment, patients will visit the clinics **4 times**. After this, visits will continue every **14 weeks** until the trial ends

*Anxiety, depression, and the risk of suicidal behavior. For further information on all assessments used in this trial, please refer to the reference below.

Wuyts WA, Richeldi L, Assassi S, Azuma A, Cottin V, Hoffmann-Vold AM, Kreuter M, Oldham JM, Martinez FJ, Valenzuela C, Wijsenbeek MS, Kanakapura M, James A, Weimann G, Drzewuski C, Coeck C, Maher TM. Design of an open-label extension trial of nerandomilast (BI 1015550) in patients with idiopathic pulmonary fibrosis and progressive pulmonary fibrosis (FIBRONEER™-ON). *BMC Pulm Med*. doi: 10.1186/s12890-025-03973-7.

ILD, interstitial lung disease, IPF, idiopathic pulmonary fibrosis; PPF, progressive pulmonary fibrosis.
