# Supplementary material for: Design of an open-label extension trial of nerandomilast (BI 1015550) in patients with idiopathic pulmonary fibrosis and progressive pulmonary fibrosis (FIBRONEER™-ON)
Source: BMC Pulm Med. 2025 Dec 4;26:10. doi: 10.1186/s12890-025-03973-7 (PMC12797674; doi:10.1186/s12890-025-03973-7)
Supplement: Supplementary file 2 — Supplementary Material 2. [file 12890_2025_3973_MOESM2_ESM.docx]

**Supplement**

**Supplementary Table 1. Further inclusion and exclusion criteria**

| **Further inclusion criteria** |
| --- |
| - WOCBP^a^ must be ready and able to use highly effective methods of birth control |
| **Further exclusion criteria** |
| - Patient not compliant in parent trial (FIBRONEER™-IPF or FIBRONEER™-ILD) with trial medication or trial visits, according to investigator’s judgement - Patients not expected to comply with the protocol requirements or not expected to complete the trial as scheduled (e.g., chronic alcohol or drug abuse or any other condition that, in the investigator’s opinion, makes the patient an unreliable trial participant) - Women who are pregnant, breast-feeding, or who plan to become pregnant while in the trial - Previous enrollment in this trial - Participation in another interventional trial |

IPF, idiopathic pulmonary fibrosis; ILD, interstitial lung disease; WOCBP, woman of childbearing potential.

^a^Woman is considered of childbearing potential, i.e., fertile, following menarche and until becoming post-menopausal unless permanently sterile. Permanent sterilization methods include hysterectomy, bilateral salpingectomy, and bilateral oophorectomy. Tubal ligation is not a method of permanent sterilization. A postmenopausal state is defined as no menses for 12 months without an alternative medical cause.

**Supplementary Table 2. Further endpoints**

| **Further endpoints** |
| --- |
| - Annual rate of decline in FVC (mL/year) over the duration of the trial - Time to absolute decline in FVC % predicted of >5% from baseline over the duration of the trial - Time to absolute decline in FVC % predicted of >5% from baseline or death over the duration of the trial - Time to relative decline in FVC % predicted of >5% from baseline or death over the duration of the trial - Time to death over the duration of the trial - Time to first hospitalization for respiratory cause over the duration of the trial - Time to first acute IPF/PPF exacerbation over the duration of the trial - Absolute change from baseline in Living with Pulmonary Fibrosis (L-PF) Symptoms Dyspnea domain score over time - Absolute change from baseline in Living with Pulmonary Fibrosis (L-PF) Symptoms Cough domain score over time - Absolute change from baseline in Living with Pulmonary Fibrosis (L-PF) Symptoms Fatigue domain score over time |

FVC, forced vital capacity; L-PF, Living with Fibrosis Symptoms and Impact questionnaire.
